# Supplementary figures and images for: Effects of cladribine on intrathecal and peripheral B and plasma cells
Source: Clin Exp Immunol. 2024 Dec 12;219(1):uxae116. doi: 10.1093/cei/uxae116 (PMC11748000; doi:10.1093/cei/uxae116)

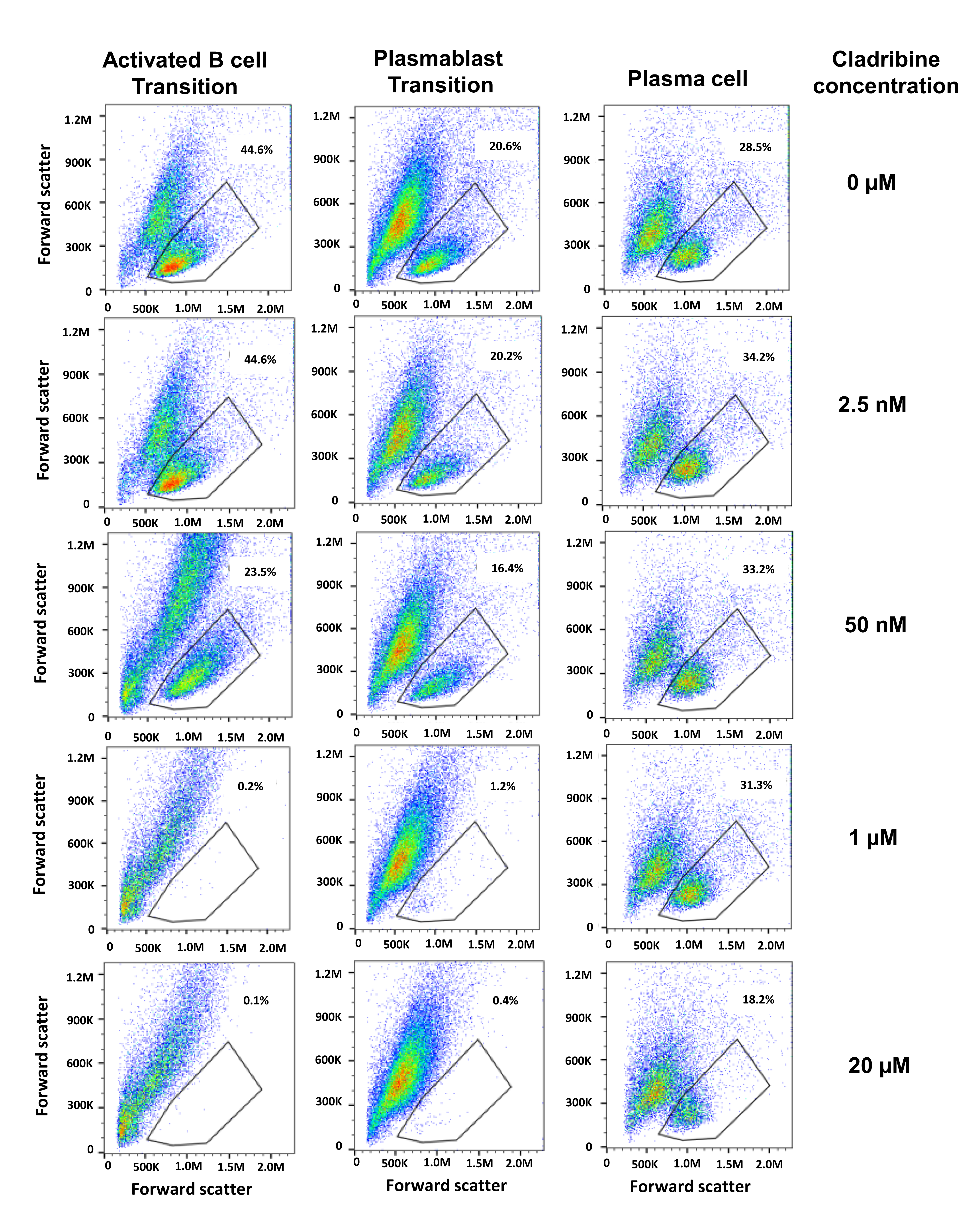

Supplement: uxae116_suppl_Supplementary_Figure_S1 [file uxae116_suppl_Supplementary_Figure_S1.png]

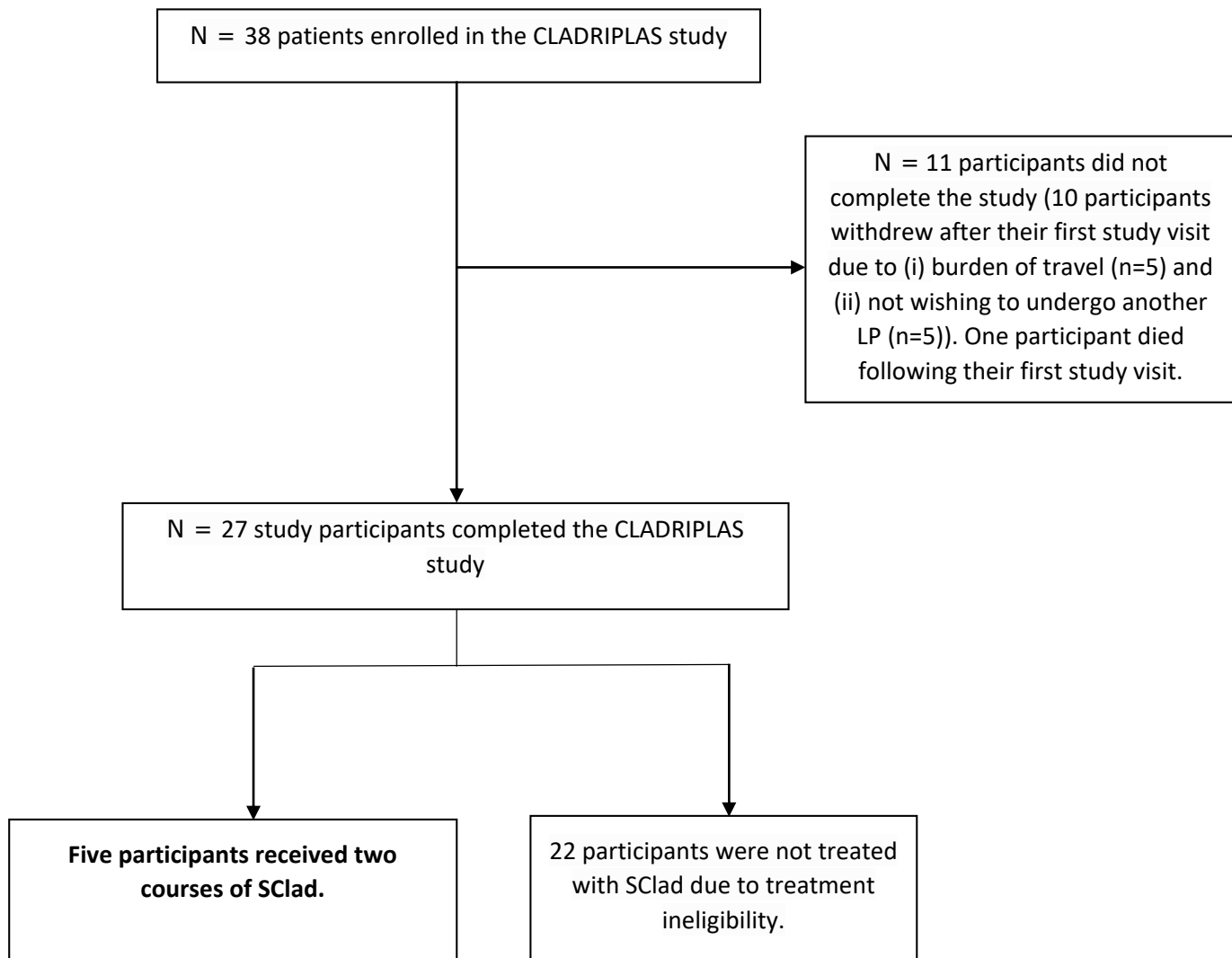

Supplement: uxae116_suppl_Supplementary_Figure_S2 [file uxae116_suppl_Supplementary_Figure_S2.pdf]
